# Associations of sarcopenia components with physical activity and nutrition in Australian older adults performing exercise training

**DOI:** 10.1186/s12877-021-02212-y

**Published:** 2021-04-26

**Authors:** Ewelina Akehurst, David Scott, Juan Peña Rodriguez, Carol Alonso Gonzalez, Jasmaine Murphy, Helen McCarthy, Sandor Dorgo, Alan Hayes

**Affiliations:** 1 Institute for Health and Sport, Victoria University, Footscray, Victoria Australia; 2 Institute for Physical Activity and Nutrition, School of Exercise and Nutrition Sciences, Deakin University, Burwood, Victoria Australia; 3 Department of Medicine, School of Clinical Sciences at Monash Health, Monash University, Clayton, Victoria Australia; 4 Australian Institute for Musculoskeletal Science (AIMSS), St Albans, Victoria Australia; 5 Faculty of Physiotherapy, The National University of Colombia, Bogota, Colombia; 6 College of Health Sciences, University of Texas at El Paso, El Paso, TX USA; 7 Department of Medicine–Western Health, Melbourne Medical School, The University of Melbourne, St Albans, Victoria Australia

**Keywords:** Helsinki University research, Nutrition, Older adults, Sarcopenia, Resistance training

## Abstract

**Background:**

The risk of progressive declines in skeletal muscle mass and strength, termed sarcopenia, increases with age, physical inactivity and poor diet. The purpose of this study was to explore and compare associations of sarcopenia components with self-reported physical activity and nutrition in older adults participating in resistance training at Helsinki University Research [HUR] and conventional gyms for over a year, once a week, on average.

**Methods:**

The study looked at differences between HUR (*n* = 3) and conventional (*n* = 1) gyms. Muscle strength (via handgrip strength and chair stands), appendicular lean mass (ALM; via dual energy X-ray absorptiometry) and physical performance (via gait speed over a 4-m distance, short physical performance battery, timed up and go and 400-m walk tests) were evaluated in 80 community-dwelling older adults (mean ± SD 76.5 ± 6.5 years). Pearson correlations explored associations for sarcopenia components with self-reported physical activity (via Physical Activity Scale for the Elderly [PASE]) and nutrition (via Australian Eating Survey).

**Results:**

No differences in PASE and the Australian Recommended Food Score (ARFS) were observed between HUR and conventional gyms, however HUR gym participants had a significantly higher self-reported protein intake (108 ± 39 g vs 88 ± 27 g; *p* = 0.029) and a trend to have higher energy intake (9698 ± 3006 kJ vs 8266 ± 2904 kJ; *p* = 0.055). In both gym groups, gait speed was positively associated with self-reported physical activity (r = 0.275; *p* = 0.039 and r = 0.423; *p* = 0.044 for HUR and conventional gyms, respectively). ALM was positively associated with protein (*p* = 0.047, r = 0.418) and energy (*p* = 0.038, r = 0.435) intake in the conventional gym group. Similar associations were observed for ALM/h^2^ in the HUR group. None of the sarcopenia components were associated with ARFS in either gym group.

**Conclusion:**

Older adults attending HUR and conventional gyms had similar self-reported function and nutrition (but not protein intake). Inadequate physical activity was associated with low gait speed and inadequate nutrition and low protein ingestion associated with low lean mas, even in older adults participating in exercise programs. Optimal physical activity and nutrition are important for maintaining muscle mass and function in older adults.

**Supplementary Information:**

The online version contains supplementary material available at 10.1186/s12877-021-02212-y.

## Background

The risk of progressive decline in skeletal muscle mass and strength, termed sarcopenia, increases with age, chronic disease, physical inactivity and poor diet [1–3]. Nutrition is an important part of muscle mass and function [4–6]. Since muscle function is affected by poor nutrition, hand grip strength (HGS) has become a marker of nutritional status [7–9] and an outcome predictor for nutritional interventions [9]. HGS is a key component of major sarcopenia definitions (the Foundation for the National Institutes of Health Sarcopenia Project and European Working Group on Sarcopenia in Older People, January 2019 update [EWGSOP2]). HGS has also been correlated with a number of performance measures, including the timed up and go (TUG) test [10], which predicts changes in functional balance [11] and was introduced as part of EWGSOP2 as a measure of sarcopenia severity. Likewise, the 400 m walk test was introduced within the EWGSOP2 definition to assess mobility and endurance, in conjunction with chair stands (strength) and the well-established sarcopenia component gait speed (GS) [12].

Low protein and energy intakes are linked to sarcopenia [5, 13] and benefits of appropriate nutrition have been reported alone and in conjunction with resistance training [14, 15]. Although the provision of exercise programs in aged-care centres is not uncommon [16–18], there is a lack of data on the relationship between sarcopenia components (muscle strength, lean mass and physical performance) and physical activity levels and nutritional status amongst participants using Helsinki University Research (HUR) and conventional gym equipment.

Our aim was to examine and compare associations of sarcopenia components with self-reported physical activity and nutrition in older adults performing exercise training at HUR and conventional gyms. We hypothesized self-reported physical activity and nutrition would be associated with muscle mass and function, with no differences according to the type of gym being used (HUR or conventional gym training).

## Methods

We applied a cross-sectional design using convenience sampling to observe participants (range 61–91 years of age) that were undergoing training exercises under supervision of exercise physiologists and physiotherapists at four gyms of Uniting AgeWell in Melbourne, Australia. The study looked at differences between HUR (*n* = 3) and conventional (*n* = 1) gyms.

### Participants

#### Sample size

The recruitment for this study came from a pool of approximately 720 existing gym members plus those willing to join the gyms during the study. Using GPower v. 3.1 [19], with muscle mass as the primary end point and an expected 20% variation around the mean, detecting a 10% difference with alpha of 0.05 and beta 0.2 would require *n* = 67. As mass was the slowest measure to change, this number ensured the ability to identify training-based adaptations in physical function. However, it was planned to recruit as many Uniting AgeWell gym users as were willing to participate by displaying posters in the four participating gyms.

#### Inclusion criteria and recruitment

The only inclusion criteria were that the subjects had to be Uniting AgeWell clients who were already gym members or had just joined the gym and were living at home or in Uniting AgeWell residential care. Participants had been assessed as able to exercise safely by Uniting AgeWell staff prior to this study commencing. Thus, there were no specific exclusion criteria as all gym clients who were accepted to take part in the Uniting AgeWell exercise training were eligible to participate, independent of type of training, frequency or duration. All participants that were available for testing were included to maintain the power for the study, thus no specific adherence criteria were required.

### Training protocol

Three sites, Forest Hill, Noble Park (both attached to the residential care) and Oakleigh gyms used HUR equipment, while the fourth site in Hawthorn used the conventional equipment. HUR gyms used HUR equipment, which was developed in Finland in 1989 and uses innovative pneumatic technology and computerised smart card and smart touch systems that record clients’ visits and work-outs [20, 21]. The Forest Hill and Oakleigh gyms included HUR Active Line equipment, such as pulleys, leg presses, hip abduction/adduction machines, leg flexion/extension machines, chest presses, rhomboid machines, trunk flexion/extension machines and iBalance and NuStep machines. Noble Park, which was the most recently opened facility, had the Premium Line equipment, including an abdominal/back roller and optimal rhomb (seated device to exercise upper body optimised for older adults). The conventional gym in Hawthorn used standard equipment, such as dumbbells, barbells, kettlebells, TheraBands, steps, medicine balls, treadmills, exercise bikes, an elliptical cross trainer and a cable weight machine. Training duration was generally 1 h, and the frequency varied depending on individual programs (usually once or twice per week), with programs ranging 2–3 sets with 8–20 repetitions.

### Sarcopenia components

#### Appendicular lean mass (ALM)

Dual-energy X-ray absorptiometry (DXA) (Hologic Horizon A, MeasureUp, Melbourne) was used to measure weight, ALM (kg), which is defined as the sum of lean soft-tissue mass from both the arms and legs [22] and a stadiometer (Charder HM200P, Charder Electronic Co. Ltd., Tachung City, Taiwan) to measure height. Absolute and normalised parameters were reported, as changes in lean mass and body size may affect loss of muscle mass with age [23].

#### Hand grip strength (HGS)

HGS (kg) was tested with subjects seated upright, with elbow bent 90° and forearm resting on an armrest support, using a handgrip dynamometer (Jamar Plus+, SI Instruments, Adelaide, Australia). Following a practice test, two trials were recorded for each hand with the subject squeezing as hard as possible and the highest score of all six tests was used for analysis [24].

#### Short physical performance battery (SPPB)

The SPPB was used to assess lower extremity function in older adults [25]. It consisted of balance with different stances, GS timed over a 4-m course at normal speed, and a five-chair stand (CS) test. The practice attempt was a single CS without a stopwatch. Participants were asked to fold their arms across chest and stand up from a chair once. If successful, five rises as fast as possible were timed from the first sitting position to the end of the fifth stand. Time was recorded using a sports stopwatch (cat. no. XC027, Jaycar, Melbourne, Australia).

#### Timed up and go (TUG)

Mobility, balance and agility were tested via the TUG (s) test at normal speed, which consisted of rising from seated position, walking three metres to a cone, turning around it, walking back and sitting down on the chair again. Participants walked at normal speed and the chair was positioned with back against a wall for safety. Following an initial trial, two further attempts were recorded and the shortest time was reported in the study [26].

#### 400-m walk

Mobility and cardiovascular fitness were assessed with a 400 m walk test (min). The standard course is 20 m with participants walking up around a cone and back 10 times as fast as possible. Due to constraints of available space, the course was 10 m long walked 20 times. Only one attempt at this test was allowed at the end of the testing day.

### Self-reported physical activity status

Physical activity status over the past week (not including any gym sessions) was assessed via a 12-item Physical Activity Scale for the Elderly (PASE) questionnaire, including activities such as walking and light, moderate or strenuous sport over the previous week [27]. Total PASE scores were calculated by multiplying the amount of time spent on each activity by respective weights and adding up all activities, usually ranging between 0 and 360, with higher scores signifying higher physical activity levels [27]. Written permission was obtained to use PASE in this study.

### Self-reported nutritional status

Participants were also asked to complete the Australian Eating Survey (AES) for adults, providing a comparison of food and nutritional intake with nutrition targets in the past 3–6 months. In this study, we analysed the Australian Recommended Food Score (ARFS), protein and energy intake derived from the AES. The ARFS has been validated for children [28–30] and adults [30]. However, this is its first use in relation to sarcopenia. The ARFS is a summary score of the overall healthiness and nutritional quality of usual eating patterns. According to the report provided ‘Guidance on Food and Nutrition Intake Output’ (2016, v.1.0), the total ARFS is 73 points, which is made up of vegetables (21), fruit (12), protein foods: meat/flesh (7), protein foods: meat/flesh alternatives (6), grains, breads, cereals (13), dairy (11), water (1), and extras (2). A score < 33 points indicates ‘needs work’, 33–38 ‘getting there’, 39–46 ‘excellent’, and 47 and over ‘outstanding. Thus, a higher ARFS score means healthier eating patterns and dietary intake that is of higher nutritional quality. Use of AES requires a paid subscription for each participant.

### Statistical analysis

Data is expressed as mean (SD) and frequency (%) unless otherwise specified. Descriptive statistics were performed on continuous variables and frequency analyses on nominal variables. Continuous data was assessed for normality and parametric (independent-sample t-tests) or non-parametric (Mann-Whitney) tests were used as appropriate. Pearson correlations examined associations for sarcopenia components (muscle strength, lean mass and physical performance) with self-reported physical activity and nutritional status. The Pearson coefficient was interpreted as weak (0.1–0.3), moderate (0.3–0.7) and strong (0.7–1.0). A *p*-value < 0.05 at 95% confidence intervals was considered statistically significant. All analyses were performed using IBM SPSS Statistics for Mac, version 25 (IBM Corp., Armonk, NY, USA).

## Results

### Baseline characteristics

**Figure 1:**
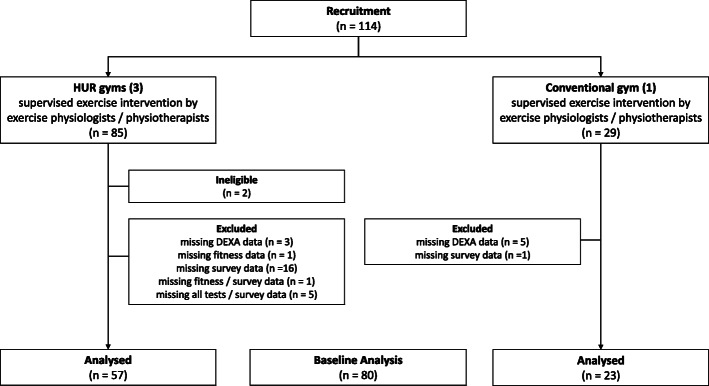
Fig. 1 Study Profile

Between February and March 2019, 114 subjects were recruited in four participating gyms. Given 20 participants were required per gym, Noble Park and Oakleigh achieved the target and the Forest Hill exceeded by 13 and Hawthorn by 11. Data from 80 community-dwelling older adults, who had already been undergoing resistance training (for a little over a year, once a week on average) was collected in March–May 2019. The study profile is presented in Fig. 1.

Comparisons of baseline characteristics between HUR and conventional gym training are shown in Table 1. Three-quarters of the cohort were of White European/Australian origin, showing no significant difference between gym groups. A subgroup analysis was not possible due to small numbers in most categories. The SPPB and PASE scores showed this was a high functioning group. There was no significant difference in PASE between the gym groups but HUR gym participants had a significantly higher protein intake than the conventional gym participants. Similarly, no significant difference was observed in ARFS between the gyms, but the HUR group tended (*p* = 0.055) to have higher self-reported energy intake than the conventional group. The only significant difference in function was the HUR group took significantly longer to complete five chair stands (*p* = 0.024) compared to the conventional gym group. This helps to explain the tendency for a lower SPPB score in the HUR group (*p* = 0.06), although both gym results are close to maximum SPPB scores, so well above values linked with poor outcomes.

**Table 1:** Comparison of baseline characteristics between HUR and conventional gym training (*n* = 80)

Characteristic		HUR (*n* = 57)	Conventional (*n* = 23)	*P*-value for difference
Demographics	Age (yr), mean (SD)	76 (6)	78 (7)	0.236
	Women, frequency (%)	37 (65)	16 (70)	0.690
	White European/Australian origin, frequency (%)	48 (84)	21 (91)	0.404
Training	Years trained, mean (SD)	1.20 (0.64)	1.39 (0.63)	0.235
	Weekly gym visits, mean (SD)	1.08 (0.54)	0.99 (0.42)	0.503
Anthropometric measurements	Height (cm), mean (SD)	163.9 (9.5)	163.2 (9.5)	0.765
	Weight (kg), mean (SD)	77.1 (17.9)	71.0 (15.4)	0.155
	BMI (kg/m^2^), mean (SD)	28.65 (6.02)	26.46 (3.76)	0.055
Muscle strength	HGS (kg), mean (SD)	26.54 (8.68)	25.47 (7.38)	0.718
	CS (s), mean (SD)	9.93 (3.98)	9.02 (3.48)	0.024 *
Lean mass	ALM (kg), mean (SD)	19.43 (5.33)	18.12 (4.81)	0.310
FNIH	ALM/_BMI_ (kg/m^2^), mean (SD)	0.69 (0.17)	0.68 (0.15)	0.945
EWGSOP2	ALM/h^2^ (kg/m^2^), mean (SD)	7.14 (1.42)	6.70 (1.11)	0.195
Physical performance	GS (m/s), mean (SD)	1.31 (0.27)	1.36 (0.17)	0.355
	SPPB (score), median (IQR)	11 (2)	12 (0)	0.060
	TUG (s), mean (SD)	8.96 (4.62)	7.41 (1.14)	0.052
	400 m walk (min), mean (SD)	5.58 (1.79)	5.34 (1.21)	0.307
Self-reported physical performance	PASE (score), mean (SD)	127 (61)	128 (44)	0.946
Self-reported nutrition	ARFS (score), mean (SD)	36 (10)	36 (8)	0.978
	AES protein (g), mean (SD)	108 (39)	88 (27)	0.029 *
	AES energy (kJ), mean (SD)	9698 (3006)	8266 (2904)	0.055

All data are mean (SD) or frequency (%). *HUR* Helsinki University Research, *BMI* body mass index, *BMC* bone mineral content, *HGS* hand grip strength, *CS* chair stand, *ALM* appendicular lean mass, *HGS* hand grip strength, *GS* gait speed, *SPPB* short physical performance battery, *TUG* timed up and go, *FNIH* Foundation for the National Institutes of Health Sarcopenia Project, *EWGSOP2* European Working Group on Sarcopenia in Older People (January 2019 update), *PASE* Physical Activity Scale for the Elderly; *AES* Australian Eating Survey, *ARFS* Australian Recommended Food Score (obtained from the *AES*). Analyses are independent sample t-tests for normally distributed data and Mann-Whitney tests for non-normally distributed data; * *p* < 0.05

Due to gender-specific cut-offs for muscle strength (HGS) and mass (ALM, ALM/BMI, ALM/h^2^), a comparison by gender is demonstrated in Table 2. As expected, both muscle strength and mass were significantly higher in men than women (*p* < 0.001). There were no differences between the gyms.

**Table 2:** Comparison of muscle strength and lean mass between HUR and conventional gym training by gender at baseline (*n* = 80)

		HUR Men (*n* = 20)	Conventional Men (*n* = 7)	*P*-value for difference	HUR Women (*n* = 37)	Conventional Women (*n* = 16)	*P*-value for difference
Muscle strength	HGS (kg)	34.04 (8.56)	32.19 (7.19)	0.588	22.49 (5.52)	22.53 (5.38)	0.979
Lean mass	ALM (kg)	23.93 (4.15)	23.84 (4.18)	0.960	16.99 (4.22)	15.61 (2.25)	0.128
FNIH	ALM/_BMI_ (kg/m^2^)	0.86 (0.14)	0.88 (0.08)	0.789	0.59 (0.10)	0.60 (0.08)	0.774
EWGSOP2	ALM/h^2^ (kg/m^2^)	7.96 (0.84)	7.97 (0.86)	0.991	6.69 (1.47)	6.15 (0.66)	0.072

*HUR* Helsinki University Research, *HGS* hand grip strength, *ALM* appendicular lean mass, *BMI* body mass index, *FNIH* Foundation for the National Institutes of Health Sarcopenia Project, *EWGSOP2* European Working Group on Sarcopenia in Older People (January 2019 update). Analyses are independent sample t-tests for normally distributed data and Mann-Whitney tests for non-normally distributed data

No significant differences were observed for self-reported physical activity or ARFS between men and women. Energy intake was significantly higher in men compared to women in both HUR (*p* = 0.001) and conventional gyms (*p* = 0.014), with similar higher protein intake in men at the HUR gyms (*p* = 0.019). No differences between the gyms were observed for either men or women for self-reported physical activity, ARFS or energy intake (Table 3), although protein intake tended to be lower in the conventional compared to the HUR gyms (*p* = 0.090 and 0.059 for men and women, respectively).

**Table 3:** Comparison of self-reported function and nutrition between HUR and conventional gym training by gender at baseline (*n* = 80)

		HUR Men (*n* = 20)	Conventional Men (*n* = 16)	*P*-value for difference	HUR Women (*n* = 37)	Conventional Women (*n* = 16)	*P*-value for difference
Self-reported physical activity	PASE (score)	139 (84)	129 (36)	0.695	121 (45)	127 (49)	0.645
Self-reported nutrition	ARFS (score)	38 (11)	37 (6)	0.760	34 (9)	35 (9)	0.756
	AES protein (g)	124.56 (42.23)	103 (19)	0.090	99.28 (34.99)	82 (28)	0.059
	AES energy (kJ)	11,409 (2963)	10,429 (2106)	0.359	8773 (2630)	7320 (2731)	0.083

*HUR* Helsinki University Research, *PASE* Physical Activity Scale for the Elderly, *AES* Australian Eating Survey, *ARFS* Australian Recommended Food Score. Analyses are independent sample t-tests for normally distributed data and Mann-Whitney tests for non-normally distributed data

### Associations of sarcopenia components with self-reported physical activity and nutritional status

For HUR gym participants, Pearson associations showed that GS had a significant weak, positive relationship with PASE, indicating that a higher GS (better function) was associated with a higher PASE score (greater self-reported physical activity levels) (Table 4). ALM had no significant correlations, however ALM/h^2^ had a significant weak, positive association with protein intake and moderate, positive association with energy intake, indicating that higher ALM/h^2^ was associated with a greater self-reported protein/energy intake. No other significant associations were observed in HUR gym participants.

**Table 4:** Associations of self-reported sarcopenia risk, physical activity, HRQoL and nutrition with sarcopenia components at baseline for HUR gym participants (*n* = 57)

		Muscle strength		Lean mass			Physical performance			
Component		HGS (kg)	CS (s)	ALM (kg)	ALM/_BMI_ (kg/m^2^)	ALM/h^2^ (kg/m^2^)	GS (m/s)	SPPB (score)	TUG (s)	400 m walk (min)
PASE (score)	Pearson Coefficient	0.220	0–.078	−0.005	−0.203	0.153	0.275^*^	0.210	−0.239	− 0.077
	*p*	0.101	0.565	0.968	0.131	0.257	**0.039**	0.117	0.073	0.567
AES-ARFS (total score)	Pearson Coefficient	−0.190	−0.025	0.097	−0.142	0.230	−0.096	− 0.183	0.178	− 0.067
	*p*	0.156	0.854	0.475	0.292	0.085	0.475	0.173	0.184	0.618
AES protein (g)	Pearson Coefficient	0.004	−0.044	0.131	−0.168	0.291^*^	0.216	0.047	−0.012	−0.109
	p	0.977	0.743	0.330	0.213	**0.028**	0.107	0.729	0.929	0.420
AES energy (kJ)	Pearson Coefficient	0.046	−0.067	0.132	−0.255	0.358^**^	0.085	−0.063	0.071	−0.071
	*p*	0.734	0.621	0.329	0.055	**0.006**	0.531	0.644	0.598	0.599

*HUR* Helsinki University Research, *HGS* hand grip strength, *CS* chair stand, *ALM* appendicular lean mass, *BMI* body mass index, *SPPB* short physical performance battery, *TUG* timed up and go, *PASE* Physical Activity Scale for the Elderly, *AES* Australian Eating Survey, *ARFS* Australian Recommended Food Score. All analyses are Pearson correlations; ** *p* < 0.01, * *p* < 0.05

For conventional gym participants, GS also had a significant moderate, positive correlation with PASE (Table 5). The 400 m walk had a significant moderate negative relationship with PASE, implying that a lower 400 m walk time (faster walking speed) was associated with a higher PASE score (greater physical activity levels). ALM had a significant moderate, positive correlation with self-reported protein and energy intake, indicating that low lean mass is associated with low protein/energy intake. When ALM was normalised for height squared, it maintained its significant moderate, positive relationship with protein and energy intake. Consistent with HUR gym participants, there was no significant association when ALM was corrected for BMI in conventional gym participants (see Tables 3 and 4, respectively). At both HUR and conventional gyms, no significant relationship was observed either for muscle strength or lean mass measures with PASE, or muscle strength and physical performance with protein/energy intake. ARFS was not associated with any of the sarcopenia components.

**Table 5:** Associations of self-reported physical activity and nutrition with sarcopenia components at baseline for conventional gym participants (*n* = 23)

		Muscle strength		Lean mass			Physical performance			
Component		HGS (kg)	CS (s)	ALM (kg)	ALM/_BMI_ (kg/m^2^)	ALM/h^2^ (kg/m^2^)	GS (m/s)	SPPB (score)	TUG (s)	400 m walk (min)
PASE (score)	Pearson Coefficient	0.396	−0.283	0.171	− 0.034	0.226	0.423^*^	0.240	−0.351	−0.479^*^
	*p*	0.061	0.190	0.435	0.879	0.301	**0.044**	0.270	0.101	**0.021**
AES-ARFS (total score)	Pearson Coefficient	−0.011	−0.097	0.007	−0.018	0.003	0.060	0.144	−0.190	−0.144
	*p*	0.959	0.660	0.973	0.934	0.989	0.786	0.512	0.386	0.512
AES protein (g)	Pearson Coefficient	0.259	0.090	0.418^*^	0.093	0.425^*^	−0.169	−0.077	0.088	0.048
	*p*	0.232	0.683	**0.047**	0.673	**0.043**	0.440	0.727	0.689	0.827
AES energy (kJ)	Pearson Coefficient	0.134	0.119	0.435^*^	0.013	0.482^*^	−0.169	−0.068	0.104	0.091
	*p*	0.542	0.588	**0.038**	0.955	**0.020**	0.440	0.756	0.637	0.679

*HGS* hand grip strength, *CS* chair stand, *ALM* appendicular lean mass, *BMI* body mass index, *SPPB* short physical performance battery, *TUG* timed up and go, *PASE* Physical Activity Scale for the Elderly, *AES* Australian Eating Survey, *ARFS* Australian Recommended Food Score. All analyses are Pearson correlations; ** *p* < 0.01, * *p* < 0.05

## Discussion

In this study, only GS (but not muscle strength) at both HUR and conventional gyms was positively associated with PASE scores, indicating higher self-reported activity is associated with better GS. This is similar to previous reports of PASE scores being positively correlated with GS [27], underlining the importance of maintaining physical activity additional to any gym sessions. Our results are inconsistent with evidence that low PASE scores, indicative of low physical activity, are related to muscle strength in older adults [31], as we did not find any association between muscle strength and PASE. It is likely that if baseline data was available, associations with strength might have been stronger, but due to participants already taking part in resistance exercise training programs designed to improve muscle strength, these associations were missing.

Rizzoli et al. [32] report that associations between self-reported and performance-based measures range from small to medium, with GS and CS among the most responsive performance-based measures. Only in the HUR gym group, 400 m walk was negatively correlated with PASE, indicating that higher levels of physical activity are associated with faster walking speeds and better endurance. Given the above, PASE appears to be a useful survey tool for correlation with measures within lower leg mobility/speed, and GS and 400 m walk continue to be positively influenced by physical activity additional to concurrent resistance training.

In our whole sample, the PASE mean score of ~ 127 was higher than reported for US (M: 103) [27], Malaysian (M: 95) [33] or Turkish community-dwelling older adults (M: 122) [34]. A higher PASE score of our cohort indicate that our participants are not only community-dwellers, but have been undertaking resistance training for over a year on average. Those who attend gyms should be encouraged to not view it as their only form of exercise, but ensure it is in addition to their regular physical activity. A recent study asked 103 Australians aged 50–92 years about sustainable lifestyles [35]. Thirty percent regarded exercise as a priority; of which 11% mentioned irregular activities (e.g., gardening and walking), another 11% purposeful exercise (e.g., gym and water aerobics) and 8% regular exercise (e.g., golf and tennis) [35]. Boulton-Lewis et al. argue that lack of awareness of exercise benefits and barriers are not new, emphasising the importance of measuring motivation and engagement to develop strategies to enhance physical activity.

None of the sarcopenia components were associated with the ARFS total score. There is limited literature on relationship between ARFS and muscle mass and function. Past research shows that a higher ARFS is linked with higher intakes of micronutrients and lower percentage energy from total and saturated fat in middle-aged populations [36]. Based on a total score of 73 for the ARFS [30], our mean results of ~ 36 for both gym groups were only slightly higher than that reported in middle-aged Australian women (M: 33), suggesting that although their diet could be improved, they may be outperforming younger ages [37]. Indeed, 33 and 39% of HUR and conventional gyms, respectively, achieved ARFS 39 and over, which is higher than that reported (21%) of middle-aged Australian women with ARFS over 40 [37]. Our higher ARFS results in two-thirds of both gym participants imply healthier eating patterns and higher diet quality.

Studies show that low protein and energy intake is linked with sarcopenia [5, 13]. There is also a strong correlation between lean mass and nutritional status in older populations [38]. Lower ALM/h^2^ was significantly associated with lower self-reported protein and energy intake in both gym groups, supporting that sufficient energy intake, and protein specifically, is essential for skeletal muscle maintenance. Our results do not show any correlations for self-reported protein/energy intake with HGS. This is inconsistent with prior research showing that since muscle function is affected by poor nutrition, HGS has become a marker of nutritional status [7–9] and an outcome predictor for nutritional interventions [9].

Again, given our participants are specifically training for strength, this may have masked any effect of poor nutrition or lower protein intakes. The HUR group had significantly higher self-reported protein intake, which may be related to the fact that they also tended to have higher BMI than the conventional gym participants. The most recent national data (2011–2012) state protein intake for the general population is around 98 g/day and 86 g/day for men in the 51–70 and 71+ age groups, respectively, and for women it is 78 g/day and 73 g/day, respectively [39]. Thus, our results suggest that our cohort consumed more protein than average, especially the HUR gym group. It is possible that participants are not obtaining as great a benefit from engaging in exercise than they would if their protein intakes were higher or of better quality (not quantity) and timed more appropriately. It is very well established, at least in younger individuals, that ingesting high-quality protein with training augments the beneficial effects [40–42]. However, older individuals require higher amounts of protein to increase protein synthesis at the same levels as a younger individual [43–45], and the recommended dietary intake (RDI) is based on not becoming deficient, rather than being an optimal dose. Nowson and O’Connell [46] report that 1.0 to 1.2 g/kg/day dietary protein is recommended for older adults or even more for those exercising and physically active to reduce muscle loss with age. A recent meta-analysis reports that muscle mass increase required protein intakes of up to 1.6 g/kg/day and was more effective in resistance-trained people but less effective in people over 60 years [45]. Protein synthesis is higher with whey protein, which is digested quickly and lower with casein, which is digested slowly, implying that a quick protein may be more suitable for reducing protein losses in older populations [46].

Timing of protein intake in combination with exercise also needs to be considered [46]. It has been reported that community-dwelling and frail older Dutch adults with a dietary intake of 1.1 g/kg/day and 1.0 g/kg/day, respectively, consumed insufficient protein at breakfast and the frail group also consumed insufficient protein at lunch (> 30 g), which was below the intake needed for muscle protein synthesis, thus likely contributing to negative health outcomes and poorer physical activity levels [46]. As a result, there is opportunity to enhance distribution of protein consumption across the day in older populations [46]. As most participants in this study had engaged in resistance training for some time, it is likely that protein quality and timing may be affecting potential for muscle mass and function gains. Thus, guidance on improving protein quality and timing of ingestion should be provided to improve lean mass health.

It is recommended regular physical activity, in addition to existing gym-based exercises, and education on nutrition be promoted at both gyms. Practical implications are that practitioners could use strategies incorporating exercises (particularly resistance training) and appropriate nutritional advice to prevent loss of muscle mass and muscle strength. Future research should incorporate post-tests to examine effects of training.

There are some limitations in our study. Since baseline data was not available, many associations did not exist as participants may have improved their muscle mass and function with training over time. The low sample size of the conventional group and not being able to show the net effect of the interventions resulted from the cross-sectional nature. The 400 m walk test had to be modified to a 10-m course walked 20 times rather than the standard 20 m walked 10 times back and forth given available space in the gyms. Results of this study may not be generalised to the general population as subjects were limited to older exercising adults in Melbourne, Australia.

## Conclusion

Older adults attending HUR and conventional gyms had similar self-reported function and nutrition (but not protein intake). Inadequate physical activity was associated with low GS and inadequate nutrition and low protein ingestion associated with low lean mas, even in older adults participating in resistance exercise training programs. Optimal physical activity and nutrition are important for maintaining skeletal muscle mass and function in older adults.

## Supplementary Information

**Additional file 1:.**

## Data Availability

The datasets used and/or analysed during the current study are available from the corresponding author on reasonable request.

## Abbreviations

AES: Australian Eating Survey
ALM: Appendicular lean mass
ARFS: Australian Recommended Food Score
BMC: Bone mineral content
BMI: Body mass index
DXA: Dual-energy X-ray absorptiometry
EWGSOP2: European Working Group on Sarcopenia in Older People (January 2019 update)
GS: Gait speed
HGS: Hand grip strength
CS: Chair stand
PASE: Physical Activity Scale for the Elderly
RDI: Recommended Dietary Intake
SPPB: Short physical performance battery
TUG: Timed up and go
