# Supplementary material for: Associations of sarcopenia components with physical activity and nutrition in Australian older adults performing exercise training
Source: BMC Geriatr. 2021 Apr 26;21:276. doi: 10.1186/s12877-021-02212-y (PMC8077926; doi:10.1186/s12877-021-02212-y)
Supplement: Supplementary file 1 — Additional file 1:. [file 12877_2021_2212_MOESM1_ESM.docx]

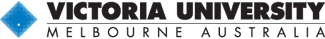


**About you**

**Date of birth: _____/_____/______ Gender:** Male Female

**­­­­­­­­** DD MM YYYY

## Physical Activity Scale for the Elderly (PASE)

PASE assesses physical activity among older adults. It has three sections: leisure time activity, household activity, and work-related activity (Note: Question 10b: 50 pounds equals 23 kg). The following 10 questions will take about 10 min to complete. Thank you.

**Please tick or write an answer where appropriate.**

### **Leisure time activity**

**1.**  **Over the past 7 days, how often did you participate in sitting activities, such as reading, watching TV or doing handcrafts?**

[0.] NEVER *(go to Question 2)*

                      [1.] SELDOM (1–2 DAYS)

                          [2.] SOMETIMES (3–4 DAYS)

                          [3.] OFTEN (5–7 DAYS)

***1.a***  **What were these activities?**

________________________________________________________________

***1.b***  **On average, how many hours did you engage in these sitting activities?**

                     [1.] Less than 1 hour

                           [2.] 1 but less than 2 hours

                         [3.] 2–4 hours

                            [4.] more than 4 hours

**2.**  **Over the past 7 days, how often did you take a walk outside your home or yard for any reason? For example, for fun or exercise, walking to work, walking the dog, etc.**

                           [0.] NEVER *(go to Question 3)*

                          [1.] SELDOM (1–2 DAYS)

                          [2.] SOMETIMES (3–4 DAYS)

                          [3.] OFTEN (5–7 DAYS)

***2a.***  **On average, how many hours per day did you spend walking?**

                        [1.] Less than 1 hour

                           [2.] 1 but less than 2 hours

                         [3.] 2–4 hours

                            [4.] more than 4 hours

**3.**  **Over the past 7 days, how often did you engage in light sport or recreational activities, such as bowling, golf with a cart, shuffleboard, fishing from a boat or pier or other similar activities?**

                          [0.] NEVER *(go to Question 4)*

                          [1.] SELDOM (1–2 DAYS)

                        [2.] SOMETIMES (3–4 DAYS)

                          [3.] OFTEN (5–7 DAYS)

***3.a***  **What were these activities?**

________________________________________________________________

***3.b***  **On average, how many hours did you engage in these light sport or recreational activities?**

                      [1.] Less than 1 hour

                           [2.] 1 but less than 2 hours

                         [3.] 2–4 hours

                            [4.] more than 4 hours

**4.**  **Over the past 7 days, how often did you engage in moderate sport and recreational activities, such as doubles tennis, ballroom dancing, hunting, ice skating, golf without a cart, softball or other similar activities?**

                          [0.] NEVER *(go to Question 5)*

                           [1.] SELDOM (1–2 DAYS)

                            [2.] SOMETIMES (3–4 DAYS)

                            [3.] OFTEN (5–7 DAYS)

***4.a***  **What were these activities?**

________________________________________________________________

***4.b***  **On average, how many hours did you engage in these moderate sport or recreational activities?**

                    [1.] Less than 1 hour

                           [2.] 1 but less than 2 hours

                         [3.] 2–4 hours

                            [4.] more than 4 hours

**5.**  **Over the past 7 days, how often did you engage in strenuous sport and recreational activities, such as jogging, swimming, cycling, singles tennis, aerobic dance, skiing (downhill or cross-country) or other similar activities?**

                            [0.] NEVER *(go to Question 6)*

                            [1.] SELDOM (1–2 DAYS)

                           [2.] SOMETIMES (3–4 DAYS)

                            [3.] OFTEN (5–7 DAYS)

***5.a***  **What were these activities?**

________________________________________________________________

***5.b***  **On average, how many hours did you engage in these strenuous sport or recreational activities?**

                          [1.] Less than 1 hour

                           [2.] 1 but less than 2 hours

                         [3.] 2–4 hours

                            [4.] more than 4 hours

**6.**  **Over the past 7 days, how often did you do any exercises specifically to increase muscle strength and endurance such as lifting weights or push-ups, etc?**

                [0.] NEVER *(go to Question 7)*

                          [1.] SELDOM (1–2 DAYS)

                        [2.] SOMETIMES (3–4 DAYS)

                          [3.] OFTEN (5–7 DAYS)

***6.a***  **What were these activities?**

________________________________________________________________

***6.b***  **On average, how many hours did you engage in these strenuous sport or recreational activities?**

                            [1.] Less than 1 hour

                           [2.] 1 but less than 2 hours

                         [3.] 2–4 hours

                            [4.] more than 4 hours

**Household activity**

**7.**  **During the past 7 days, have you done any light housework, such as dusting or washing dishes?**

[1.] NO [2.] YES

**8.**  **During the past 7 days, have you done any heavy housework or chores, such as vacuuming, scrubbing floors, washing windows, or carrying wood?**

[1.] NO [2.] YES

**9. During the past 7 days, did you engage in any of the following activities? Please answer YES or NO for each item.**

|  | NO | YES |
| --- | --- | --- |
| 1. Home repairs like painting, wallpapering, electrical work, etc. | 1 | 2 |
| 1. Lawn work or yard care, including snow or leaf removal, wood chopping, etc. | 1 | 2 |
| 1. Outdoor gardening | 1 | 2 |
| 1. Caring for another person, such as children, dependent spouse, or another adult | 1 | 2 |

### **Work-related activity**

**10.**  **During the past 7 days, did you work for pay or as a volunteer?**

[1.] NO *(End of survey)*  [2.] YES

***10a.***  **How many hours per week did you work for pay and or as a volunteer?**

____ hours

***10b.***  **Which of the following categories best describes the amount of physical activity required on your job and or volunteer work?**

[1.] Mainly sitting with some slight arm movement **[Examples:** office worker, watchmaker, seated assembly line worker, bus driver, etc.]

[2.] Sitting or standing with some walking [**Examples:** cashier, general office worker, light tool and machinery worker]

[3.] Walking with some handling of materials generally weighing less than 50 pounds **[Examples:** mailman, waiter/waitress, construction worker, heavy tool and machinery worker]

[4.] Walking and heavy manual work often requiring handling of materials weighting over 50 pounds **[Examples:** lumberjack, stone mason, farm or general labourer]

**Thank you for your time completing this survey!**

Note. Reprinted from “The Physical Activity Scale for the Elderly (PASE): development and evaluation,” by R. A. Washburn, K. W. Smith, A. M. Jette, C. A. & Janney, 1993, Journal of clinical epidemiology, 46(2), 153–162. <https://doi.org/10.1016/0895-4356(93)90053-4>. Reprinted with permission.
